# Outcomes of isolated mitral valve surgery performed via right anterolateral thoracotomy: a single-centre experience

**DOI:** 10.3389/fcvm.2025.1625773

**Published:** 2025-08-05

**Authors:** Liang Huang, Yue-Hang Yang, Chi-Yang Xie, Si Chen, Chao Guo, Jia-Wei Shi

**Affiliations:** Department of Cardiovascular Surgery, Union Hospital, Tongji Medical College, Huazhong University of Science and Technology, Wuhan, Hubei, China

**Keywords:** mitral valve surgery, right anterolateral thoracotomy, median sternotomy, thoracic and pulmonary complications, outcomes, follow-up

## Abstract

**Objectives:**

The evidence underlying thoracic and pulmonary complications and clinical outcomes after isolated mitral valve surgery performed via right anterolateral thoracotomy is inconclusive. This study retrospectively compared the postoperative thoracic and pulmonary complications and clinical outcomes of isolated mitral valve surgery performed via right anterolateral thoracotomy vs. median sternotomy.

**Methods:**

Patients data undergoing isolated mitral valve surgery in our institution were analyzed. Propensity score matching was applied to minimize differences between patients undergoing right anterolateral thoracotomy and median sternotomy. Intraoperative parameters, postoperative thoracic and pulmonary complications, and other postoperative outcomes were compared. Outpatient follow-ups were conducted.

**Results:**

Of 711 individuals who met study criteria, 298 underwent right anterolateral thoracotomy and 413 underwent median sternotomy surgery. Propensity score matching resulted in 279 matched pairs for further analysis. Patients’ characteristics were comparable in the matched cohorts. The right anterolateral thoracotomy group had higher incidences of subcutaneous emphysema (23.3% vs. 2.9%, *P* < 0.001), pneumothorax (12.5% vs. 2.5%, *P* < 0.001), right diaphragmatic elevation (7.5% vs. 0.7%, *P* < 0.001), and massive pleural effusion (3.6% vs. 1.1%, *P* = 0.049) compared to the median sternotomy group. However, the right anterolateral thoracotomy group had lower 24-hour postoperative drainage [median (IQR), 200 (110, 350) vs. 300 (230, 415), ml; *P* < 0.001], postoperative red blood cell and plasma transfusion volume [median (IQR), 7.0 (4.0, 11.0) vs. 10.0 (5.5, 17.0), U; *P* < 0.001], ICU stay duration [median (IQR), 2 (2, 3) vs. 2 (2, 3), day; *P* = 0.004], and postoperative days [median (IQR), 9 (8, 11) vs. 12 (10, 15), day; *P* < 0.001]. Follow-up data of patients in two groups had no significant differences (*P* > 0.05). Multivariable logistic regression analysis revealed that incision type, age, atrial fibrillation, and coronary heart disease were significant factors influencing postoperative thoracic and pulmonary complications and course (*P* < 0.05).

**Conclusions:**

Isolated mitral valve surgery via right anterolateral thoracotomy was associated with more thoracic and pulmonary complications and shorter 24-hour postoperative drainage, ICU stay duration and postoperative days compared with median sternotomy, which potentially related to the choice of incision site, specific patient conditions, and surgical techniques.

## Introduction

Traditionally, mitral valve surgery is performed via a median sternotomy (ST). However, mitral valve surgery using small incisions was often not considered in the past due to challenges in exposure and inferior outcomes (1). The rapid development and refinement of techniques over the past decade have led to the realization that a minimally invasive approach enables valve surgery to be performed with results, at the very least, equivalent to those of conventional valve surgery done in experienced centers (2, 3). In 1996, Carpentier et al. (4) performed the first video-assisted mitral valve repair through a minithoracotomy using ventricular fibrillation. With more experiences, video-assisted, 2-dimensional endoscopes and robotics were introduced by Carpentier et al. and Chitwood et al. (4–6). Compared to traditional incisions, minimal mitral valve surgery significantly reduces skin incisions, preserves the anatomical integrity of the thoracic cage, and minimizes the surgical trauma to patients. Minimal mitral valve surgery has now evolved into a safe, efficient treatment option providing a lower incidence of new-onset arrhythmia and prolonged intubation, shorter hospital duration, greater satisfaction, and less morbidity (7–9). However, a study of mitral valve repair confirmed that minithoracotomy is not superior to sternotomy in recovery of physical function at 12 weeks (3). Minimal invasive procedures have emerged as the preferred approach in many medical centres, although they continue to be a subject of ongoing debate (10). Minimal mitral valve surgery at our center has been rapidly developing, with a large number of patients undergoing small incision procedures each year. However, the associated complications and clinical outcomes require further investigation. Based on this context, we retrospectively analyzed isolated mitral valve surgery performed at our institution, comparing the postoperative thoracic and pulmonary complications (TPCs) and outcomes of right anterolateral thoracotomy (RAT) vs. ST, with the aim of summarizing our center's experience in mitral valve surgery performed via RAT.

## Methods

### Study population

Patients undergoing isolated mitral valve surgery in our center from January 2020 to December 2023 were retrospectively analyzed. Inclusion criteria were as follows: age ≥18 years; New York Heart Association (NYHA) functional class Ⅱ–Ⅳ before surgery; confirmed mitral valve disease via echocardiography or other examinations meeting surgical indications; and isolated mitral valve repair or replacement surgery. Exclusion criteria included infective endocarditis, coronary artery disease requiring concurrent coronary artery bypass grafting, morbid obesity (body mass index ≥30 kg/m^2^), and emergency surgery.

### Outcomes and sources of data

The outcomes of this study included intraoperative data and postoperative data. Intraoperative data included duration of surgery, aortic cross-clamp time (ACCT), and cardiopulmonary bypass (CPB) time. Postoperative data included the TPCs (including subcutaneous emphysema, pneumothorax, right diaphragmatic elevation, atelectasis, rib fracture, pneumonia, pulmonary edema) and other postoperative outcomes including 24 h postoperative drainage, blood transfusion (referring to red blood cell and plasma transfusion), massive pericardial effusion, duration of intubation, ICU stay duration, postoperative days, reoperation for bleeding, cerebral infarction, poor wound healing or wound infection and in-hospital mortality. All patients go through six months follow up. Primary outcomes of follow up included adverse cardiovascular events (including arrhythmia, heart failure), endocarditis, perivalvular leakage, valvular insufficiency, valve thrombosis, 3-month readmission rate, and all-cause mortality. Secondary outcomes of follow up included poor wound healing, pneumonia, pleural effusion, and pericardial effusion. Data on patient demographics were obtained. Data above were obtained from our institutional databases.

### Surgical techniques

**Figure 1:**
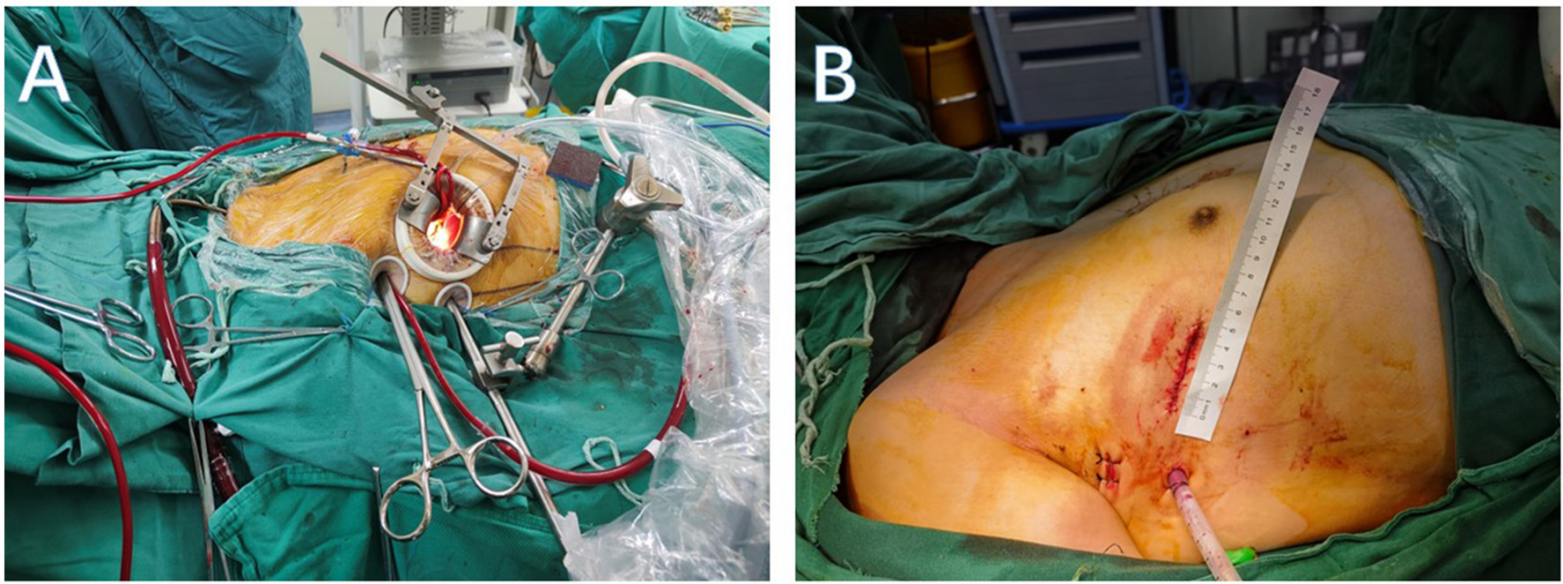
**(A)** Intraoperative incision and auxiliary hole. **(B)** Postoperative incision and drainage hole.

The minimal mitral valve surgery in this study referred to the right anterolateral thoracotomy, with and without endoscopic assistance. For male patients, a 6–8 cm incision was made through the fourth intercostal space along the anterior axillary line, and for female patients, the incision was made below the right breast along the same intercostal space (Figure 1). A 2 cm groin incision was made for femoral artery and vein cannulation to establish CPB. For thoracoscopic assistance, the fourth or fifth intercostal space along the anterior axillary line was used for camera insertion.

### Statistical analysis

Statistical analysis was performed using SPSS 25.0. Propensity score matching was performed using a 1:1 nearest neighbor matching algorithm without replacement with distances determined by logistic regression. Propensity score matching was performed based on the following variables: sex, age, body mass index (BMI), left ventricular anteroposterior diameter (LVAD), left ventricular ejection fraction (LVEF), hypertension, pneumonia, chronic obstructive pulmonary disease (COPD), atrial fibrillation, cerebral infarction, coronary heart disease, and degree of valvular disease. The optimal matching algorithm was used as a sensitivity analysis. Continuous variables were expressed as mean ± standard deviation (*x* ± *s*) or as medians with interquartile ranges, and comparisons were made using rank-sum tests. Categorical variables were expressed as percentages, and comparisons were made using chi-square tests. A *P*-value < 0.05 was considered statistically significant.

## Results

### Characteristics of patients

Characteristics of patients by surgical approach before and after propensity score matching were shown in Table 1. Of 711 individuals who met study criteria, 298 underwent RAT surgery and 413 underwent ST surgery. The median (IQR) age in the RAT group was 55.0 (44.0, 63.0) years and in the ST group was 55.0 (48.0, 61.0) years. The RAT group had a higher preoperative LVEF (%) compared to the ST group [median (IQR), 65 (62, 68) vs. 65 (60, 68); *P* = 0.022] and significantly lower BMI [median (IQR), 22.8 (20.8, 25.0) vs. 23.5 (21.1, 25.9); *P* = 0.017]. Other parameters showed no significant differences between the groups (*P* > 0.05). Propensity score matching resulted in 279 matched pairs for further analysis, between which baseline characteristics and preoperative data showed no significant differences (*P* > 0.05).

**Table 1:** Characteristics of patients by surgical approach before and after propensity score matching.

Variables	Before PSM					After PSM				
	Total (*n* = 711)	RAT (*n* = 298)	ST (*n* = 413)	*P*	SMD	Total (*n* = 558)	RAT (*n* = 279)	ST (*n* = 279)	*P*	SMD
Age, M (Q₁, Q₃)	55.0 (47.0, 62.0)	55.0 (44.0, 63.0)	55.0 (48.0, 61.0)	0.705	0.046	55.0 (46.0, 62.0)	55.0 (44.0, 63.0)	55.0 (47.0, 61.0)	0.808	−0.006
Male, *n* (%)	372 (52.3)	158 (53.0)	214 (51.8)	0.751	−0.024	291 (52.2)	150 (53.8)	141 (50.5)	0.446	−0.065
BMI, M (Q₁, Q₃)	23.2 (21.0, 25.6)	22.8 (20.8, 25.0)	23.5 (21.1, 25.9)	0.017	−0.122	23.0 (21.0, 25.4)	22.8 (21.1, 25.1)	23.3 (21.0, 25.6)	0.302	0.116
LVAD, M (Q₁, Q₃)	5.2 (4.7, 5.6)	5.2 (4.8, 5.6)	5.2 (4.7, 5.7)	0.992	0.059	5.2 (4.7, 5.6)	5.2 (4.8, 5.6)	5.2 (4.7, 5.7)	0.923	0.061
LVEF, M (Q₁, Q₃)	65 (61, 68)	65 (62, 68)	65 (60, 68)	0.022	−0.257	65 (62, 68)	65 (62, 68)	65 (62, 69)	0.346	0.048
Hypertension, *n* (%)	104 (14.6)	37 (12.4)	67 (16.2)	0.156	0.103	74 (13.3)	36 (12.9)	38 (13.6)	0.803	0.021
Pneumonia, *n* (%)	33 (4.6)	12 (4.0)	21 (5.1)	0.508	0.048	25 (4.5)	11 (3.9)	14 (5.0)	0.539	0.049
COPD, *n* (%)	47 (6.6)	21 (7.1)	26 (6.3)	0.691	−0.031	38 (6.8)	21 (7.5)	17 (6.1)	0.501	−0.06
Atrial fibrillation, *n* (%)	21 (3.0)	6 (2.0)	15 (3.6)	0.208	0.087	12 (2.2)	6 (2.2)	6 (2.2)	1	0
Cerebral infarction, *n* (%)	49 (6.9)	23 (7.7)	26 (6.3)	0.46	−0.059	35 (6.3)	19 (6.8)	16 (5.7)	0.6	−0.046
Coronary heart disease, *n* (%)	43 (6.1)	19 (6.4)	24 (5.8)	0.755	−0.024	29 (5.2)	17 (6.1)	12 (4.3)	0.34	−0.088
Degree of valvular disease, *n* (%)				0.347					0.956	
Mild	10 (1.4)	2 (0.7)	8 (2.0)		0.092	4 (0.7)	2 (0.7)	2 (0.7)		0
Moderate	61 (8.6)	27 (9.1)	34 (8.2)		−0.03	50 (9.0)	24 (8.6)	26 (9.3)		−0.025
Severe	640 (90.0)	269 (90.2)	371 (89.8)		−0.014	504 (90.3)	253 (90.7)	251 (90.0)		0.024

Measures are expressed as median (IQR), and counts are expressed as *n* (%); Rank-sum tests were used to compare continuous variables between groups, while categorical variables were analyzed using the chi-square test. PSM, propensity score matching; RAT, right anterolateral thoracotomy; ST, median sternotomy; SMD, standardized mean difference; BMI, body mass index; LVAD, left ventricular anteroposterior diameter; LVEF, left ventricular ejection fraction; COPD, chronic obstructive pulmonary disease.

### Intraoperative and postoperative data

Intraoperative and postoperative data were summarized in Table 2 and Table 3, respectively. The results showed that RAT group had significantly longer CBP time [median (IQR), 141 (116, 169) vs. 95 (76, 114), min; *P* < 0.001], operation time [median (IQR), 270 (240, 330) vs. 217 (180, 266), min; *P* < 0.001], and ACCT [median (IQR), 90 (74, 114) vs. 59 (45, 74), min; *P* < 0.001] compared to ST group. TPCs including subcutaneous emphysema (23.3% vs. 2.9%; *P* < 0.001), pneumothorax (12.5% vs. 2.5%; *P* < 0.001), right diaphragmatic elevation (7.5% vs. 0.7%; *P* < 0.001), and massive pleural effusion (3.6% vs. 1.1%; *P* = 0.049) were more frequent in RAT group, while other TPCs including atelectasis (13.3% vs. 17.6%; *P* = 0.159), rib fracture (0.7% vs. 0%; *P* = 0.479), pneumonia (30.8% vs. 34.8%; *P* = 0.321), pulmonary edema (1.4% vs. 3.2%; *P* = 0.161) had no differences. However, RAT group had lower 24-hour postoperative drainage [median (IQR), 200 (110, 350) vs. 300 (230, 415), ml; *P* < 0.001], perioperative blood transfusion [median (IQR), 7.0 (4.0, 11.0) vs. 10.0 (5.5, 17.0), U; *P* < 0.001], ICU stay duration [median (IQR), 2 (2, 3) vs. 2 (2, 3), day; *P* = 0.004], and postoperative days [median (IQR), 9 (8, 11) vs. 12 (10, 15), day; *P* < 0.001].There were no significant differences between the groups in duration of intubation [median (IQR), 1.0 (1.0, 1.0) vs. 1.0 (1.0, 1.0), day; *P* = 0.226] and in-hospital mortality (0.4% vs. 0.7%; *P* = 1). Other postoperative outcomes including massive pericardial effusion (0.7% vs. 1.1%; *P* = 1), reoperation for bleeding (1.1% vs. 0.7%; *P* = 1), poor wound healing or wound infection (0.4% vs. 0%; *P* = 1) and cerebral infarction (0.4% vs. 1.1%; *P* = 0.616) showed no significant differences between the groups.

**Table 2:** Intra-operative data by surgical approach in the subgroups paires by propensity score.

Variables	Total (*n* = 558)	RAT (*n* = 279)	ST (*n* = 279)	*P*
CPB time, M (Q₁, Q₃)	115 (90, 149)	141 (116, 169)	95 (76, 114)	<.001
ACCT, M (Q₁, Q₃)	74 (55, 99)	90 (74, 114)	59 (45, 74)	<.001
Operation time, M (Q₁, Q₃)	249 (210, 300)	270 (240, 330)	217 (180, 266)	<.001

Measures are expressed as median (IQR). Rank-sum tests were used to compare continuous variables between groups. RAT, right anterolateral thoracotomy; ST, median sternotomy; CPB, cardiopulmonary bypass, ACCT, aortic cross-clamping time.

**Table 3:** TPCs and postoperative course in the subgroups paires by propensity score.

Variables	Total (*n* = 558)	RAT (*n* = 279)	ST (*n* = 279)	*P*
TPCs				
Subcutaneous emphysema, *n* (%)	73 (13.1)	65 (23.3)	8 (2.9)	<.001
Pneumothorax, *n* (%)	42 (7.5)	35 (12.5)	7 (2.5)	<.001
Right diaphragm elevation, *n* (%)	23 (4.1)	21 (7.5)	2 (0.7)	<.001
Massive pleural effusion, *n* (%)	13 (2.3)	10 (3.6)	3 (1.1)	0.049
Atelectasis, *n* (%)	86 (15.4)	37 (13.3)	49 (17.6)	0.159
Rib fracture, *n* (%)	2 (0.4)	2 (0.7)	0 (0.0)	0.479
Pneumonia, *n* (%)	183 (32.8)	86 (30.8)	97 (34.8)	0.321
Pulmonary edema, *n* (%)	13 (2.3)	4 (1.4)	9 (3.2)	0.161
Postoperative course				
24 h postoperative drainage (ml), M (Q₁, Q₃)	250 (200, 400)	200 (110, 350)	300 (230, 415)	<.001
Blood transfusion (U), M (Q₁, Q₃)	8.0 (4.1, 13.5)	7.0 (4.0, 11.0)	10.0 (5.5, 17.0)	<.001
Massive pericardial effusion, *n* (%)	5 (0.9)	2 (0.7)	3 (1.1)	1
Reoperation for bleeding, *n* (%)	5 (0.9)	3 (1.1)	2 (0.7)	1
Duration of intubation (day), M (Q₁, Q₃)	1.0 (1.0, 1.0)	1.0 (1.0, 1.0)	1.0 (1.0, 1.0)	0.226
ICU stay duration (day), M (Q₁, Q₃)	2 (2, 3)	2 (2, 3)	2 (2, 3)	0.004
Postoperative days, M (Q₁, Q₃)	10 (8, 13)	9 (8, 11)	12 (10, 15)	<.001
Poor wound healing or wound infection, *n* (%)	1 (0.2)	1 (0.4)	0 (0.0)	1
Cerebral infarction, *n* (%)	4 (0.7)	1 (0.4)	3 (1.1)	0.616
In-hospital mortality, *n* (%)	3 (0.5)	1 (0.4)	2 (0.7)	1

Measures are expressed as mean ± SD and median (IQR), and counts are expressed as *n* (%); Rank-sum tests were used to compare continuous variables between groups, while categorical variables were analyzed using the chi-square test. TPCs, thoracic and pulmonary complications; RAT, right anterolateral thoracotomy; ST, median sternotomy; ICU, intensive care unit.

### Logistic regression analysis and subgroup analysis

Univariable logistic regression analysis indicated that incision type (*P* < 0.001), age (*P* < 0.001), coronary heart disease (*P* = 0.045) and atrial fibrillation (*P* = 0.01) were significant factors between the two groups. Other variables, including sex, BMI, LVAD, LVEF, hypertension, pneumonia, COPD, cerebral infarction, and severity of valvular lesions showed no significant differences (*P* > 0.05). Multivariable logistic regression analysis indicated that incision type, age, coronary heart disease and atrial fibrillation were key factors influencing postoperative complications (*P* < 0.05) (Table 4).

**Table 4:** Univariable and multivariable logistic regression for the postoperative complications.

Variables	Model 1	*P*-value	Model 2	*P*-value	Model 3	*P*-value
	OR (95% CI)		OR (95% CI)		OR (95% CI)	
Surgical incision	0.37 (0.25∼0.55)	<0.001	0.35 (0.23∼0.53)	<0.001	0.33 (0.21∼0.50)	<0.001
Males	1.36 (0.92∼2.00)	0.118	1.18 (0.78∼1.80)	0.414	1.10 (0.69∼1.77)	0.679
Age	1.03 (1.01∼1.05)	<0.001	1.03 (1.01∼1.05)	0.001	1.03 (1.02∼1.06)	<0.001
BMI	1.04 (0.98∼1.11)	0.15	1.04 (0.97∼1.10)	0.258	1.06 (0.99∼1.13)	0.091
LVAD	1.16 (0.88∼1.52)	0.293			1.10 (0.80∼1.51)	0.554
LVEF	0.98 (0.95∼1.02)	0.35			0.98 (0.94∼1.02)	0.34
Hypertension	0.94 (0.54∼1.65)	0.84			0.77 (0.41∼1.45)	0.427
Pneumonia	1.33 (0.49∼3.61)	0.576			1.01 (0.33∼3.05)	0.986
COPD	1.81 (0.74∼4.44)	0.192			2.64 (0.98∼7.08)	0.054
Atrial fibrillation	0.45 (0.14∼1.44)	0.18			0.28 (0.08∼0.96)	0.043
Cerebral infarction	0.95 (0.43∼2.07)	0.889			0.65 (0.27∼1.55)	0.33
Coronary heart disease	0.61 (0.27∼1.34)	0.215			0.40 (0.17∼0.98)	0.045
Degree of valvular disease						
Mild	1.00 (Reference)				1.00 (Reference)	
Moderate	0.20 (0.02∼1.75)	0.147			0.12 (0.01∼1.12)	0.062
Severe	0.47 (0.06∼3.90)	0.488			0.31 (0.04∼2.67)	0.288

Statistical analysis method used: logistic regression analysis. Model 1 was unadjusted, Model 2 was partially adjusted (Surgical incision, Males, Age, BMI, atrial fibrillation), and Model 3 was fully adjusted (model 2 adds hypertension, pneumonia, COPD, atrial fibrillation, coronary heart disease, LVAD, LVEF, degree of valvular disease). BMI, body mass index; LVAD, left ventricular anteroposterior diameter; LVEF, left ventricular ejection fraction; COPD, chronic obstructive pulmonary disease.

**Figure 2:**
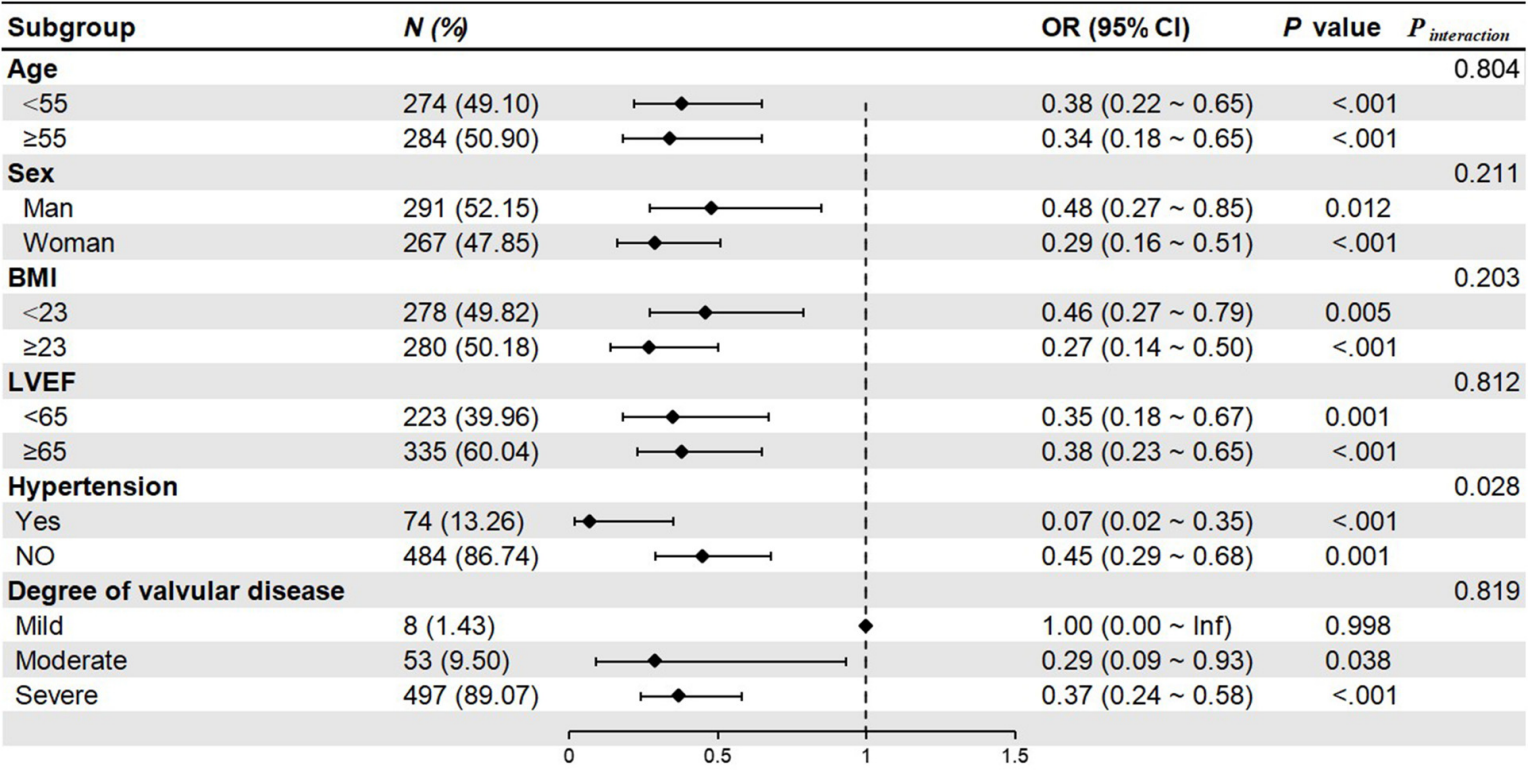
Forest plot of subgroup analysis. BMI, body mass index; LVEF, left ventricular ejection fraction.

Subgroup analysis revealed that median sternotomy was a protective factor against the TPCs. The forest plot showed that patients with hypertension could significantly benefit from median sternotomy surgery (*P* interaction = 0.025). Although other subgroups could also benefit from median sternotomy surgery (*P* < 0.05), but there were no differences by groups (*P* interaction > 0.05) (Figure 2).

### Follow-up data

Follow-up contents were shown in Supplementary Table S1. Primary outcomes included adverse cardiovascular events (including arrhythmia, heart failure), infective endocarditis, perivalvular leakage, valvular insufficiency, valve thrombosis, 3-months readmission rate, and all-cause mortality after discharge. Secondary outcomes included poor wound healing, pneumonia, pleural effusion, and pericardial effusion. No significant differences were observed between the groups (*P* > 0.05).

### Learning curve of RAT surgery

**Figure 3:**
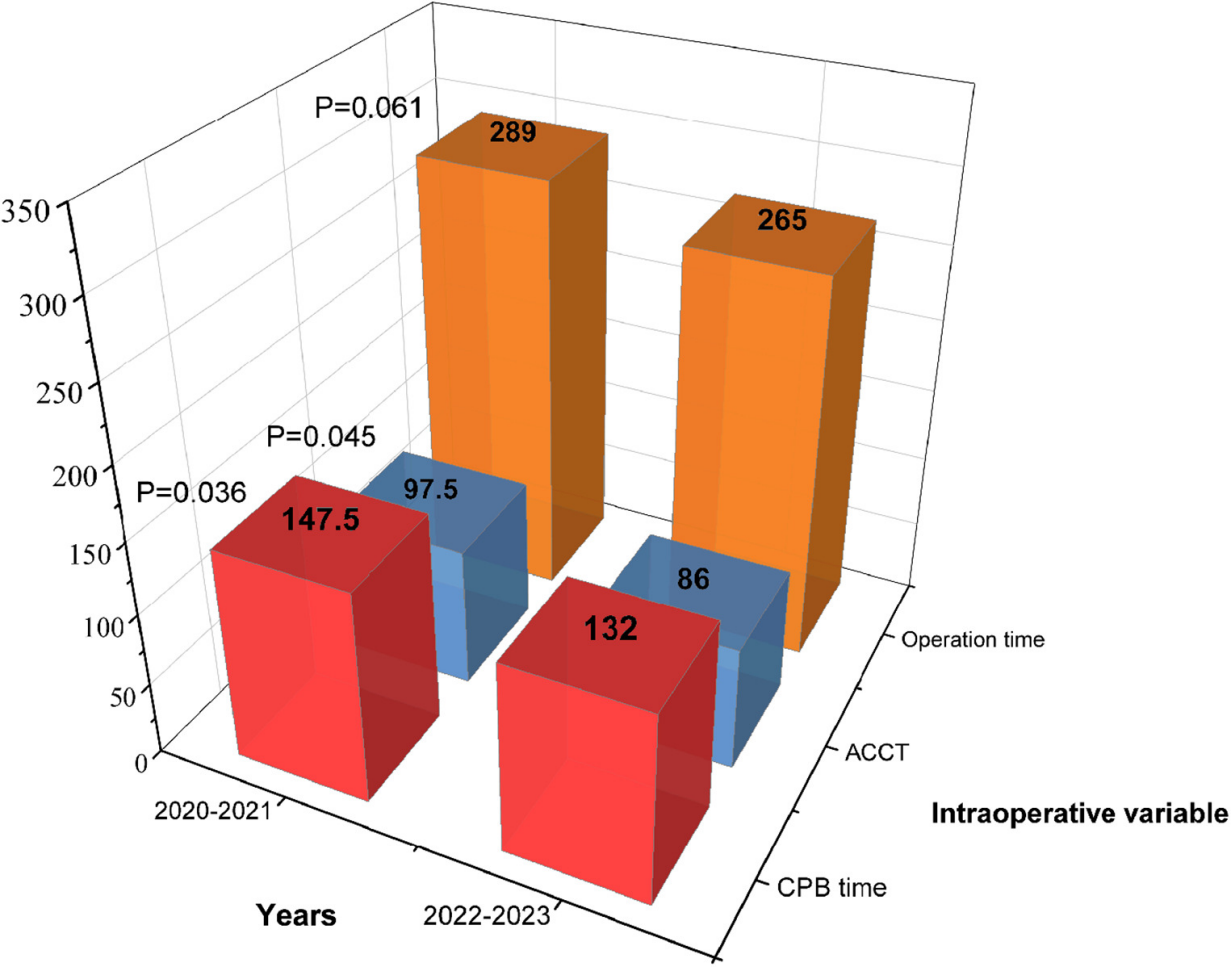
Intraoperative variables based on the surgery date in the RAT group.

**Figure 4:**
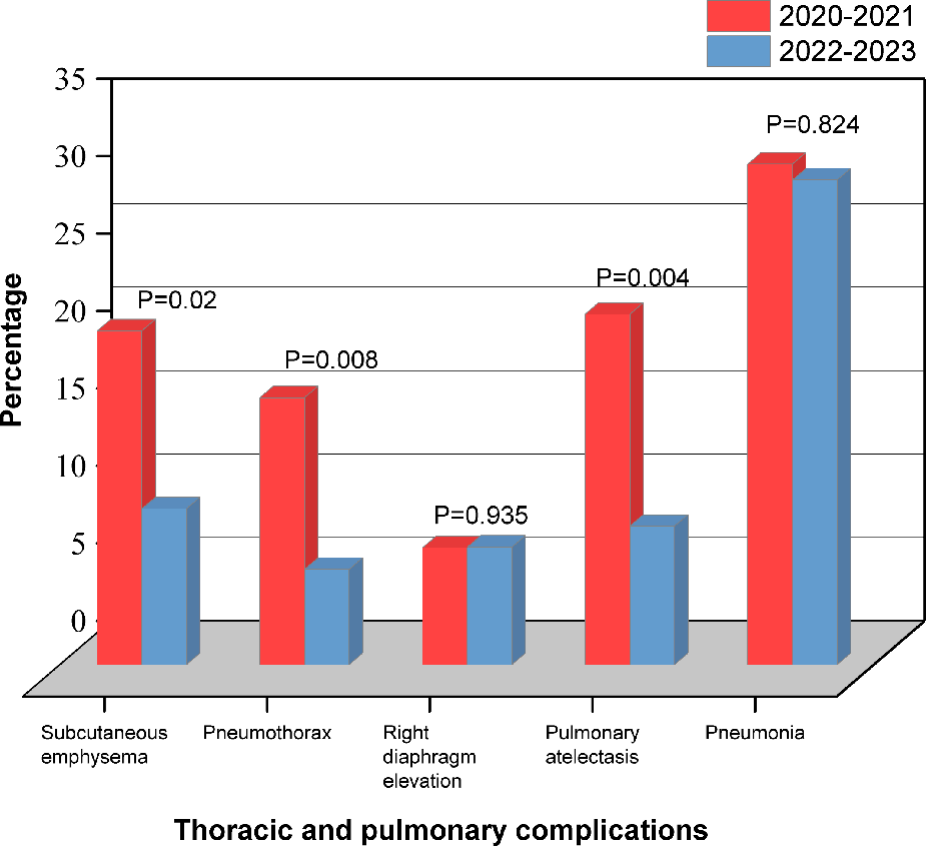
Thoracic and pulmonary complications based on the surgery date in the RAT group.

Patients in RAT group were divided into two subgroups based on the surgery date (January 2022 and beyond). Statistical analysis showed that 2021–2022 group was more in CPB time (147.5 vs. 132, min; *P* = 0.036), ACCT (97.5 vs. 86, min; *P* = 0.045) and operation time (289 vs. 265, min; *P* = 0.061) than 2022–2023 group (Figure 3). TPCs including subcutaneous emphysema (*P* = 0.02), pneumothorax (*P* = 0.008) and atelectasis (*P* = 0.004) significantly decreased in 2022–2023 group compared to 2021–2022 group. No significant differences were observed in the incidences of right diaphragmatic elevation (*P* = 0.935) and pneumonia (*P* = 0.824) (Figure 4). These findings reflect the continuous improvement of RAT techniques in our center, with a steady reduction in related TPCs over time.

## Discussion

The number of mitral valve surgery via RAT in our center has been growing over the years and is ranked among the top cardiac centers in China. This study focused on the postoperative TPCs and the outcomes of isolated mitral valve surgery via RAT. The TPCs refer to the complications occurring in the chest and lungs after mitral valve surgery. Our analysis showed that the two procedures had similar duration of intubation, reoperation for bleeding and in-hospital mortality. Compared to conventional sternotomy, the RAT group had higher incidences of subcutaneous emphysema, pneumothorax, right diaphragmatic elevation and massive pleural effusion. The RAT group reduced 24-hour postoperative drainage, postoperative red blood cell and plasma transfusion volume, ICU stay duration, postoperative days but did not affect the incidence of massive pericardial effusion, poor wound healing or wound infection, and cerebral infarction. In terms of six-months follow up, there were no significant differences observed between the groups.

Reviewing the development history of the median sternotomy entry approach for over one hundred years, on the one hand, it provides our surgical team with optimal control of the operative field, both visually and manually, on the other hand, it also has some common complications including superficial wound infection, bony nonunion/sternal instability, sternal dehiscence, and mediastinitis (11, 12). With the rapid development of medical technology, the concept of minimally invasive mitral valve surgery has emerged to reduce surgical trauma and meet patients' aesthetic demands. In terms of postoperative convalescence and thoracic stability, small incision surgery has unique advantages. Several minimally invasive techniques have since been developed (3, 5). Common minimally invasive approaches include right minithoracotomy (13), robot-assisted right thoracic approaches (14), and partial sternotomy (15). Date from the German Society for Thoracic and Cardiovascular Surgery and the American Society of Thoracic Surgeons' Adult Cardiac Surgery Database showed that the application of minimally invasive mitral valve surgery increased rapidly since 2004 (16, 17). A review showed that compared with traditional sternotomy, minimally invasive mitral valve surgery reduces intraoperative bleeding, alleviates patient pain, shortens ventilation time, and improves cosmetic outcomes (18, 19). In terms of safety, no significant differences in mortality were reported between minimally invasive and conventional approaches across multiple comparative studies (20–22).

However, some studies have reported more complications associated with minimally invasive mitral valve surgery, including neurological dysfunction, aortic complications, and groin infections (23, 24). A single-center study noted that 19.9% of patients undergoing minimally invasive mitral valve surgery had radiographic evidence of right pulmonary vascular congestion after surgery, with 5 patients requiring ECMO due to severe pulmonary complications (25). While many studies highlight the advantages of minimally invasive mitral valve surgery in reducing pain (18, 19), cases of severe incisional pain and long thoracic nerve injury leading to prolonged hospital stays and recovery have also been reported (26).

Data from 2020 to 2023 at our center indicated improvements in duration of surgery, CPB time, ACCT and decline of some TPCs. These results reflect a true learning curve in RAT. Some experts argue that the learning curve for minimally invasive mitral valve surgery should prevent its widespread implementation (17, 27). Significant inter-surgeon variability exists, and a relatively large number of cases are required to overcome the learning curve. Furthermore, Antunes MJ suggest that minimally invasive mitral valve surgery should be restricted to high-volume centers specializing in mitral valve procedures (28).

When initiating an minimally invasive mitral valve surgery program, all patients should be considered candidates unless contraindicated. However, certain comorbidities and anatomical considerations should be treated as relative contraindications (29), including: 1. Severe aortic, iliac, or femoral artery disease that impedes safe arterial retrograde perfusion; 2. Left ventricular ejection fraction <25%; 3. Severe right heart dysfunction; 4. Pulmonary artery pressure >70 mmHg; 5. Aortic diameter >4 cm (if using intra-aortic balloon pump); 6. Significant mitral annular calcification; 7. More than mild aortic regurgitation; 8. Kyphosis or pectus excavatum; 9. Morbid obesity or extreme muscularity.

The implementation of minimally invasive mitral valve surgery is a process that requires continuous learning and accumulation of experience. A complete learning curve is essential, emphasizing the importance of technical refinement. Due to the longer learning curve of RAT, its introduction should begin at high-volume cardiac centers with experienced surgeons to minimize severe complications. A patient-centered approach should be maintained, selecting appropriate patients for RAT to maximize patient benefits.

## Study limitations

This study has several limitations. First, it is a retrospective study, which would be associated with information and selection bias and a larger sample size is required to validate the results. Second, propensity score matching analysis was performed to minimize the selection bias, residual selection bias from unmeasured/unknown confounders was inevitable in the absence of randomization. Third, data on cardiac perioperative injuries and related complications were not collected, leaving room for further investigation into their impact. Fourth, as a single-centre study, it limits the generalizability of the results. Lastly, long-term follow-up data were insufficient, necessitating extended tracking to better understand the long-term outcomes and complications of RAT surgery.

## Conclusions

Isolated mitral valve surgery via RAT is safe and feasible, with similar duration of intubation, reoperation for bleeding and in-hospital mortality compared to ST. However, some of the postoperative TPCs are more frequent. Moreover, subgroup analysis revealed that patients with hypertension could significantly benefit from median sternotomy surgery. Therefore, surgeons should adhere to the learning curve of RAT surgery, thoroughly evaluate patient conditions, and carefully select incision types to ensure optimal outcomes.

## Data Availability

The original contributions presented in the study are included in the article/Supplementary Material, further inquiries can be directed to the corresponding author.
