# Supplementary material for: Outcomes of isolated mitral valve surgery performed via right anterolateral thoracotomy: a single-centre experience
Source: Front Cardiovasc Med. 2025 Aug 5;12:1625773. doi: 10.3389/fcvm.2025.1625773 (PMC12361239; doi:10.3389/fcvm.2025.1625773)
Supplement: Supplementary file 1 [file Table1.docx]

| **Supplementary table 1 Six months follow-up data of patients in two groups.** | | | | |
| --- | --- | --- | --- | --- |
| Variables | Total (n = 558) | RAT (n = 279) | ST (n = 279) | *P* |
| 3-months readmission, n (%) | 26 (4.7) | 15 (5.4) | 11 (3.9) | 0.422 |
| All-cause mortality, n (%) | 3 (0.5) | 1 (0.4) | 2 (0.7) | 1 |
| Adverse cardiovascular events, n (%) |  |  |  | 0.128 |
| Arrhythmia | 15 (2.7) | 4 (1.4) | 11 (3.9) |  |
| Heart failure | 3 (0.5) | 1 (0.4) | 2 (0.7) |  |
| Valve-related complications, n (%) |  |  |  | 0.774 |
| Infective endocarditis | 8 (1.4) | 5 (1.8) | 3 (1.1) |  |
| Perivalvular leakage | 2 (0.4) | 1 (0.4) | 1 (0.4) |  |
| Valve thrombosis | 1 (0.2) | 0 (0.00) | 1 (0.4) |  |
| Valvular insufficiency | 24 (4.3) | 14 (5.0) | 10 (3.6) |  |
| Other complications, n (%) |  |  |  | 0.89 |
| Poor incision healing | 11 (2.0) | 6 (2.2) | 5 (1.8) |  |
| Pulmonary infection | 21 (3.8) | 10 (3.6) | 11 (3.9) |  |
| Pleural effusion | 19 (3.4) | 11 (3.9) | 8 (2.9) |  |
| Pericardial effusion | 9 (1.6) | 4 (1.4) | 5 (1.8) |  |
| At least two complications | 29 (5.2) | 17 (6.1) | 12 (4.3) |  |
| Continuous variables are expressed as n (%). RAT, right anterolateral thoracotomy; ST, median sternotomy. | | | | |
